# A Comprehensive Review of Hepatitis B Vaccine Nonresponse and Associated Risk Factors

**DOI:** 10.3390/vaccines12070710

**Published:** 2024-06-25

**Authors:** Albashir Tahir, Sa’adatu Haruna Shinkafi, Ahmed Subeh Alshrari, Abdulmajeed Yunusa, Muhammad Tukur Umar, Shuaibu Abdullahi Hudu, Abdulgafar Olayiwola Jimoh

**Affiliations:** 1 Department of Pharmacology and Therapeutics, Faculty of Basic Clinical Sciences, College of Health Sciences, Usmanu Danfodiyo University, Sokoto 840001, Nigeria; albashirtahir@gmail.com (A.T.); yunusa.abdulmajeed@udusok.edu.ng (A.Y.); tukur.umar@udusok.edu.ng (M.T.U.); 2 Department of Pharmacology, Faculty of Basic Medical Sciences, Bauchi State University, Gadau 751105, Nigeria; 3 Department of Microbiology and Parasitology, Usmanu Danfodiyo University Teaching Hospital, Sokoto 23270, Nigeria; 4 Medical Laboratory Technology Department, Faculty of Applied Medical Science, Northern Border University, Arar 91431, Saudi Arabia; ahmed.alsharari@nbu.edu.sa; 5 Department of Basic Medical and Dental Sciences, Faculty of Dentistry, Zarqa University, Zarqa 13110, Jordan; 6 Department of Microbiology and Parasitology, Faculty of Basic Clinical Sciences, College of Health Sciences, Usmanu Danfodiyo University, Sokoto 840232, Nigeria

**Keywords:** hepatitis B, hepatitis B vaccine, vaccine nonresponse, nonresponders, risk factors

## Abstract

Hepatitis B virus (HBV) infection remains a significant global health concern worldwide, contributing to high rates of mortality and morbidity, including chronic hepatitis B, cirrhosis, and hepatocellular carcinoma (HCC). Universal vaccination programs have significantly reduced the rate of HBV transmission; however, a subset of individuals fail to develop a protective immune response following vaccination and are termed nonresponders. A comprehensive search strategy using the PubMed, Google Scholar, and Web of Science databases was employed to search for relevant studies using keywords including “hepatitis B vaccine”, “vaccine nonresponse”, “immunogenicity”, “immune response to the hepatitis B vaccine”, and “associated risk factors”. Factors influencing the vaccine’s response include demographic factors, such as age and sex, with increased nonresponse rates being observed in older adults and males. Obesity, smoking, and alcohol consumption are lifestyle factors that decrease the vaccine response. Medical conditions, including diabetes, chronic kidney and liver diseases, HIV, celiac disease, and inflammatory bowel disease, affect the vaccine response. Major histocompatibility complex (MHC) haplotypes and genetic polymorphisms linked to immune regulation are genetic factors that further influence the vaccine’s effectiveness. To reduce the global burden of hepatitis B infection, it is essential to understand these factors to improve vaccine effectiveness and develop individualized vaccination strategies.

## 1. Introduction

Hepatitis B is a viral infection caused by the hepatitis B virus (HBV), a DNA virus of the Hepadnaviridae family, primarily affecting the liver and causing both acute and chronic hepatic diseases. It is a major global health concern, with approximately 254 million people living with chronic HBV infection and 1.2 million new cases reported annually. In 2022 alone, about 1.1 million people died from hepatitis B-related complications, largely due to hepatocellular carcinoma (HCC) and liver cirrhosis [1]. The regions with the largest infection burden are the Western Pacific (97 million people) and Africa (65 million people), respectively. There are 61 million infected people in the Southeast Asia region, compared to 15, 11, and 5 million in the WHO Eastern Mediterranean, European, and American regions, respectively [1]. Inadequate vaccination coverage, limited access to healthcare services, vertical transmission during childbirth, and cultural practices are contributing factors [1,2]. Host-related factors (such as age, sex, and immune status), along with virological factors (such as HBV DNA levels, viral genotype, and quantitative surface antigen), affect the severity of infection [3]. While chronic infections can result in serious and potentially fatal complications, such as cirrhosis and hepatocellular carcinoma, acute cases typically present with symptoms like poor appetite, nausea, stomach pain, fatigue, and jaundice [4].

Hepatitis B vaccination remains an important tool for mitigating the disease burden associated with HBV infection by providing long-term protection by stimulating the immune system to produce antibodies against the hepatitis B virus surface antigen [5,6]. In highly endemic regions in particular, universal hepatitis B vaccination programs have significantly lowered the infection rates and prevented chronic liver disease and HCC [7]. It was the first vaccine to prevent chronic liver disease and hepatocellular carcinoma [8]. While most immunocompetent individuals acquire at least two decades of protection from primary immunization, about 5–10% of vaccine recipients, termed ‘nonresponders’, do not develop a protective anti-HBs titer following the full vaccine series [9]. This lack of response can be due to various factors, such as age, gender, BMI, immune status, genetics, or underlying medical conditions. This review aims to provide insights into the factors contributing to hepatitis B vaccine nonresponse and the underlying mechanisms.

## 2. Methodology

The search for articles was carried out in the PubMed, Google Scholar, and Web of Science databases using the following keywords: “hepatitis B vaccine”, “vaccine nonresponse”, “immunogenicity”, “immune response to the hepatitis B vaccine”, and “associated risk factors”. Boolean operators (AND/OR) were employed to combine search terms. The literature search was structured as follows: (“Hepatitis B Vaccine” OR “HBV Vaccine”) AND (“Vaccine Non-response” OR “Non-responders”) AND (“Immune Response”, “immunogenicity” OR “Immunology”) AND (“Associated Risk Factors” OR “Risk Factors”). This strategy aimed to capture all relevant studies investigating the complex interplay between the immunological response to the HBV vaccine and the factors influencing vaccine nonresponse. Titles and abstracts of the obtained results were screened for inclusion, and the full texts of relevant studies were retrieved for review. References of the included studies were scrutinized to identify additional relevant publications.

## 3. Hepatitis B Vaccine and the Immune System

Hepatitis B surface antigen (HBsAg), a protein from the outer envelope of HBV, is a significant diagnostic marker for hepatitis B infection and the primary constituent of the hepatitis B vaccine. It triggers humoral and cellular immunity by causing the immune system to produce antibodies against it [10,11]. Antigen-presenting cells, such as dendritic cells, process and present HBsAg to T-cells upon the activation of the adaptive immune system, initiating an immune response. This step is critical in resolving the infection, and most therapeutic vaccines developed to date have focused on eliciting T-cell responses specific to HBV [10,12]. Furthermore, the activation of B cells leads to the production of anti-HBs and anti-HBc antibodies. These antibodies circulate in the bloodstream and neutralize the virus upon infection. The development of memory B cells enables the immune system to retain the characteristics of HBsAg, facilitating a quick and robust immune response when the virus is subsequently encountered. The gradual decline in antibody levels is compensated for by the presence of these memory B cells, ensuring that individuals maintain protective immunity [13].

An incomplete HBV vaccine series can significantly impair the immune response and overall efficacy of the hepatitis B vaccine. The recommended HBV vaccination series consists of three doses, which are essential for developing robust and long-term immunity. Missing doses or nonadherence to the recommended schedule can result in suboptimal immune memory and reduced vaccine efficacy, compromising long-term protection against HBV [14]. Additionally, if the series is not completed, the effectiveness of the vaccine can wane over time, further increasing the risk of nonresponsiveness and potential HBV infection [14]. Different vaccination schedules, such as the 0–1–2-month and 0–1–6-month schedules, have been compared, with varying results on compliance rates and immune effects. The 0–1–2-month schedule was found to have a similar short-term immune effect and a higher rate of completion in an adult population, and it may induce better long-term immune memory compared to the 0–1–6 schedule [15]. The time since the last HBV vaccine significantly impacts the persistence of immunity. Studies have shown that antibody levels decline over time, with a negative correlation between anti-HBs titers and time [16]. The primary risk associated with a long-elapsed time since the last HBV vaccination is decreased protection against HBV infection. Protection decreases notably after 20 years since vaccination, with older individuals and those with compromised immune systems being particularly affected [17].

Following the completion of the full hepatitis B vaccine series, approximately 90–95% of individuals develop seroprotective antibody levels post-vaccination, characterized by anti-HBs levels ≥ 10 mIU/mL. However, a small percentage (around 5–10%) may not achieve the desired protective antibody levels. This nonresponse to the hepatitis B vaccine is a significant concern, and several risk factors have been identified to be associated with it, including age; sex; obesity; smoking; alcohol abuse; chronic illnesses such as diabetes mellitus, kidney disease, liver disease, and HIV; and genetic factors. The effects of these factors and possible mechanisms of eliciting the nonresponse are summarized in Table 1.

### 3.1. Age

Variations in the effectiveness of the hepatitis B vaccine have been observed with age. Studies indicate that people aged ≥ 40 years have less protection compared to younger adults, who have a higher rate of HBV protection after vaccination [18]. This is primarily due to immunosenescence, a process by which the immune system weakens with age. A meta-analysis of 24 trials revealed a strong correlation between older age and a higher risk of nonresponse to the vaccination. Compared with younger people, older individuals have a pooled relative risk of 1.76 for failing to achieve an adequate response [56]. Another meta-analysis by Yang et al. (2016) revealed a significant decrease in response to the hepatitis B vaccine among older adults [20].

Immune senescence weakens the adaptive immune response in the elderly, leading to a reduced thymic size, compromised T-cell production and differentiation, and poor antibody production. Furthermore, decreased CD4 T-cells hinder antibody production due to insufficient germinal center activation, whereas an increased loss of CD28 molecules on T-cells causes T-cell anergy and apoptosis [57]. With an advancing age, there is a deficiency in CD62L, an adhesion molecule that plays a crucial role in enabling undifferentiated and memory T-cells to migrate into lymph nodes. This deficiency contributes to senescence. Additionally, suppressed CD62L expression impairs the T-cell activation of B cells, resulting in reduced antibody formation [19].

### 3.2. Gender

Gender disparities in host responses to the hepatitis B vaccine have been revealed in several studies, with females exhibiting a higher immune response [22]. This supports the general observation that females often show stronger immune responses to vaccines than males [58]. Additionally, males appear to have a higher risk of complications from hepatitis B infection, suggesting potential gender differences in susceptibility to the virus and disease progression. This could be attributed to the contrasting effects of the sex hormones androgen and estrogen on the immune system. The stimulation of monocytes by estrogen leads to the release of IL-10, which, in turn, triggers the secretion of IgG and IgM by B cells. On the other hand, testosterone has been found to have an inhibitory effect on the production of IgG and IgM, and it suppresses IL-6 production by monocytes [21,22,59]. Variations in the distribution of immunological genes on sex chromosomes may contribute to the observed variations in vaccine response between genders as females have more immunological genes on the X chromosome than males. Furthermore, females possess more effective immune memory, which can rapidly induce high anti-HBs levels upon vaccination [60]. However, contrary to the above findings, a meta-analysis revealed a similar response to the vaccine across the sexes, with a relative risk of vaccine nonresponse of 1.40 for males in comparison to females [20].

### 3.3. Obesity

Reduced responsiveness to the hepatitis B vaccine has been associated with obesity [23,25]. Several mechanisms have been reported to account for the decreased response, including the distribution of the vaccine in fat tissue rather than muscle, the enzymatic denaturation of the vaccine, and the impaired function of antibody-secreting plasma cells [25]. A reduced response to the vaccine also occurs in obese individuals as a result of systemic inflammation caused by leptin and the inflammation of B cells, which impair T-cell responses, impede lymphocyte division and proliferation, decrease natural killer cell activity, and disrupt the balance of CD4/CD8 T-cells [61].

Obesity and non-alcoholic fatty liver disease (NAFLD) impair normal immune function. This causes the exacerbation of chronic diseases and complications of metabolic syndrome [40]. A study of HBV-naïve adults with NAFLD demonstrated significantly lower HBV vaccine-specific antibody and T-cell responses, suggesting a higher risk of nonresponse to the vaccine among obese individuals with NAFLD [40]. Obesity can increase the risk of hepatitis B vaccine-escape mutations, potentially impacting its effectiveness [25]. Vitamin D deficiency is another factor linked to decreased hepatitis B vaccine response in obese individuals. Vitamin D promotes the maturation and function of key immune cells, such as monocyte precursors, macrophages, and T-lymphocytes, as well as lowers inflammation [23]. Several factors contribute to vitamin D deficiency, including volumetric dilution in the body, its storage in adipose tissue, inadequate exposure to sunlight, and reduced synthesis in adipose tissue and the liver [24].

### 3.4. Smoking

Cigarette smoking has been reported to influence both innate and adaptive immunity, which, in turn, affects various immune cells, including B cells, T helper cells, memory T/B lymphocytes, macrophages, dendritic cells, and natural killer cells [27]. A 20-year cohort study by Fonzo et al. (2023) revealed that smoking has detrimental effects on both the immediate and long-term immune responses to hepatitis B vaccine during infancy [26]. Cigarette smoke generally impairs the immune system’s ability to fight infections; however, it paradoxically enhances Th2-mediated immune responses that drive allergic asthma [62]. Cigarette smoking not only weakens immunity against infections but also fosters a weakened immune response during prolonged chronic infection, leading to cross-reactive immunopathology. Although the exact mechanism underlying the immunopathology associated with smoking remains largely unclear, smokers are 29% more likely to have an increased non-protective antibody titer [26]. This negative impact is attributed to the damage caused by nicotine to the T-cell antigen-mediated pathway and intracellular calcium response, which, in turn, restricts the antibody-forming cell response [20].

### 3.5. Alcohol Consumption

Depending on the consumption rate, the modulatory effect of alcohol on the immune system can affect the response to the hepatitis B vaccine. An enhanced immune response with reduced inflammation has been linked to moderate consumption; however, chronic consumption leads to an impaired response to the hepatitis B vaccine due to a deranged immune system [28]. While some studies suggest that alcohol consumption, especially among females, may suppress immune responses [63], others did not find a significant variance in the response between alcohol consumers and non-consumer to the hepatitis B vaccine [20,42]. A meta-analysis of 37 articles, including 21,053 adults, did not report a significant difference in the response to the vaccine among alcohol consumers [20].

While alcohol has been known to significantly affect many components of the innate and acquired immune systems, including B cells, T-cells, neutrophils, and macrophages, the mechanism through which the nonresponse to hepatitis B occurs following alcohol consumption remains unclear [64]. The suppression of B cell function by alcohol could result in decreased antibody production against HBV antigens, which could contribute to chronic hepatitis B infection [32]. As B cells also function as antigen-presenting cells, an alcohol-induced reduction in B cell numbers could inhibit antigen presentation, further impairing the immune response [31]. Chronic alcohol consumption inhibits the responsiveness of B cells to various cytokines, particularly IL-2 and IL-4, which are essential for B cell differentiation and antibody production [30]. Additionally, enhanced viral replication, increased oxidative stress, and cytotoxicity in alcohol consumers exacerbate HBV infection [29].

### 3.6. Diabetes

The link between diabetes and the response to the hepatitis B vaccine has been the focus of numerous studies. A systematic review revealed that the seroprotection rates for individuals with diabetes who completed the hepatitis B vaccine series varied from 31.3 to 94.4%. This is in comparison to a range of 35.2–96.9% in individuals without diabetes [35]. The impaired response in individuals with diabetes has been attributed to factors including defects in antigen presentation, a decrease in the number of circulating helper T-cells, and an impaired cellular response. These factors may contribute to the less robust antibody production following hepatitis B vaccination. Furthermore, the presence of specific human leukocyte antigen alleles in individuals with diabetes has been linked to an impaired vaccine response [35]. Other studies have demonstrated an association between obesity, a major risk factor for type 2 diabetes, and a reduced response to the vaccine in adults [34]. A meta-analysis also established a clear association between diabetes and an impaired response to the vaccine in patients undergoing long-term dialysis, likely due to numerous changes in cellular and humoral immune responses observed in non-uremic patients with diabetes [33].

### 3.7. Chronic Kidney Disease (CKD)

The response rate to the hepatitis B vaccine in patients with CKD is less than that observed in the general population. This diminished response is linked to a multitude of factors, including an advanced age, the male gender, body weight, co-existing hepatitis C virus positivity, diabetes mellitus, lymphomas, inflammation, hyperparathyroidism, hypoalbuminemia, erythropoietin resistance, vitamin D deficiency, and the immunosuppressive effects of uremia and dialyzer membranes [38]. The seroconversion rates are the highest in the early stages of CKD. However, a substantial number of patients do not respond to the vaccine. Moreover, over 90% of patients with advanced CKD rarely achieve a protective seroconversion rate [65]. In patients with CKD who are not undergoing dialysis, the seroconversion rates after three doses of a 40 µg hepatitis B vaccine were 35.7%, 66.6%, and 87.5% for those with chronic, moderate, and mild CKD, respectively. These rates showed significant improvement after a fourth dose of the vaccine [36]. HBV reactive T- and B cells could be detected in patients undergoing hemodialysis regardless of their capacity to mount an optimum serological response, indicating potential protection [66]. Patients undergoing hemodialysis or peritoneal dialysis often need higher doses of the vaccine and may require additional booster doses to achieve a protective immune response. The use of third-generation vaccines, novel adjuvants, or immunostimulants for enhanced immunogenicity was also recommended [37]. The response to the vaccine diminishes over time, with seroconversion rates decreasing as renal function deteriorates [38].

### 3.8. Chronic Liver Disease (CLD)

Patients with CLD often exhibit a poor response to the hepatitis B vaccine. This blunted response can be attributed to several factors, including an advanced age, the male sex, obesity (especially prevalent in non-alcoholic fatty liver disease), diabetes mellitus, and cirrhosis [40]. In a study of patients with chronic HCV infection, only 79% responded to the hepatitis B vaccine, and cirrhosis was the sole identifiable risk factor contributing to the reduced response [42]. The seroconversion rates are variable in patients with CLD, ranging from 16% to 79%, with an overall low rate of 35% observed in a study of 126 patients [41]. Specifically, the response to the vaccine is notably reduced in patients with cirrhosis. A previous study demonstrated that patients with cirrhosis exhibited a lower vaccine responsiveness of 51% compared to 72% in the non-cirrhotic population [41]. Another study reported that seroconversion rates following the two-dose hepatitis B vaccine were 94% in patients with chronic HCV and 95% in those with CLD. However, these rates tend to decrease with liver disease severity [39].

### 3.9. Chronic Hepatitis C Virus (HCV)

Chronic HCV infection has been shown to adversely affect the immune response to hepatitis B vaccination. While some studies, such as those by Khokhar et al. (2014) and Lee et al. (1999), reported similar vaccine response rates among individuals infected with HCV and the general population [67,68], a significant number of studies have indicated a sub-optimal to a markedly lower response rate in patients with HCV [42,69,70]. In a study involving 525 patients infected with HCV, 21% did not respond to the hepatitis B vaccine [42]. Furthermore, a meta-analysis of 11 studies involving 704 patients with HCV reported that HCV infections are associated with a decreased response to the standard hepatitis B vaccination schedule [43]. The reduced response appears to be multifactorial, involving the up-regulation of a negative immune modulator for T-cell receptor signaling (PD-1) and impaired humoral immunity [71,72]. The overexpression of the killer cell lectin-like receptor sub-family G member 1 (KLRG1), an inhibitory receptor on CD4 cells, has been found to inhibit the proliferation of CD4+ cells and the secretion of IL-2 in patients infected with HCV [44]. The cytotoxic immune response is strongly inhibited in murine models [73,74]. The poor vaccine response of patients with HCV is attributed to the impairment of the humoral arm of their immune response [71]. Additionally, the presence of liver cirrhosis and diabetes were identified as risk factors for low response [75].

### 3.10. Human Immunodeficiency Virus (HIV)

About 1% of patients with hepatitis B, translating to 2.7 million people, also have HIV according to the WHO [1]. Following hepatitis B vaccination, there is considerable variation in the seroconversion rate among patients with HIV, with rates ranging from 18% to 72%, which is largely influenced by patients’ immune statuses. For those not receiving Highly Active Antiretroviral Therapy (HAART), the response rate falls between 30 and 50%. However, for patients undergoing HAART, the response rate markedly improves, reaching between 60% and 70% [46,47]. This response is directly proportional to the CD4+ count [76]. A meta-analysis demonstrated that an undetectable viral load and a high CD4+ count are significantly associated with a positive vaccine response. Higher seroconversion correlated with CD4+ levels above 500 cells/mm3. Even at CD4+ levels >350 cells/mm3, some studies have demonstrated an improved vaccine response [45,47,77]. In a study conducted in Vietnam, the response to the hepatitis B vaccine among adults living with HIV was higher in female patients (71.4%) than in male patients (56.8%) [46].

### 3.11. Celiac Disease

Celiac disease (CD) is a chronic autoimmune condition of the small intestine that results from an immune reaction to gluten. This reaction damages the villi in the small intestine, disrupting nutrient absorption, including those essential for immune function, thereby compromising the immune system and its response to vaccines. Compared with the general population, people with CD respond poorly to the hepatitis B vaccine [48,50]. Studies have reported a nonresponse rate of about 54–78% [78]. Various explanations for this reduced response have been proposed in several studies, including genetic predisposition, the existence of specific haplotypes, and gluten consumption. CD patients with disease-specific human leukocyte antigen (HLA) haplotypes HLA-B8, DR3, and DQ2 demonstrated a diminished response to the vaccine. Notably, HLA-DQ2 appears to be linked to this nonresponse. The higher rate of nonresponsiveness observed in these individuals could be attributed to this genetic predisposition [54]. Gluten intake during hepatitis B vaccination has been hypothesized to alter immune responses due to competition for HLA-DQ2 binding between gliadin peptides and HBsAg protein fragments [49]. Despite the above evidence, Zingone et al. (2013) reported that gluten exposure does not influence the hepatitis B vaccine response [55]. Trovato et al. (2021) reported an association between the absence of seroconversion to the hepatitis B vaccine and elevated IgA-antibodies against transglutaminase 2 (TGA-IgA) and an older age at diagnosis in patients with CD [79].

### 3.12. Inflammatory Bowel Disease (IBD)

IBD is a chronic gastrointestinal tract disorder comprising Crohn’s disease and ulcerative colitis. Similar to CD, malabsorption and nutritional deficiencies caused by IBD may compromise immune function; thus, an impaired vaccination response has been linked to malnutrition, which is common in patients with IBD [80]. Individuals with IBD have a poor serological response to the hepatitis B vaccine, with a pooled response rate of 61%, which is affected by the presence of immunosuppressive treatments [51,81]. Patients with IBD responded poorly to the vaccine [53]. The immunosuppressive state resulting from both the illness and its treatment is the main cause of reduced hepatitis B vaccine response in patients with IBD. Immunosuppressive therapies, such as biologics and immunomodulators, can further compromise the humoral response of the immune system to the hepatitis B vaccine by interfering with nucleic acid synthesis and inhibiting the proliferation of activated lymphocytes [52]. Additionally, studies have indicated correlations between tumor necrosis factor-alpha (TNF-α) levels and memory B cell activity, indicating its critical role in controlling memory B cell function [82]. This implies that anti-TNF-α medications may affect memory B cells, influencing immune responses and vaccination efficacy.

### 3.13. Genetic Fact

The response to the hepatitis B vaccine is greatly affected by genetic factors (Table 2). This response is dominantly inherited and associated with the major histocompatibility complex (MHC). Certain HLA haplotypes display aberrant or deficient immune responses to HBsAg, leading to nonresponsiveness in individuals with specific genetic profiles [83]. The antibody responses to the HBV S and pre-S regions are influenced by MHC-linked genes [84]. The presence or absence of specific MHC genes in responders versus nonresponders highlights the dominant role of immune response genes in HBsAg antibody formation. A linkage analysis revealed a strong correlation between MHC haplotypes and the absence of an antibody response to HBsAg, further emphasizing the genetic influence on vaccine effectiveness. Studies have demonstrated associations between specific genotypes, such as CCR5Δ32, and reduced HBV vaccine immunogenicity [85].

Several genetic variants linked to the hepatitis B vaccine immune response and susceptibility to chronic HBV infection have been identified by a multitude of genome-wide association studies (GWAS); rs1015166, rs12527394, rs2395179, rs3135363, rs3132969, rs34039593, rs35953215, rs3830066, rs4248166, rs477515, rs7745040, rs7770370, rs9267665, rs9268202, rs9268831, rs9273062, rs9277176, rs9277356, rs9277535, rs9277464, and rs9277549 are notable genetic variants linked to the vaccine’s response [86]. Genetic polymorphisms related to HLA and interleukins can affect the response to the vaccine in both positive and negative ways. There has been evidence linking specific haplotypes of HLA-DQ and HLA-DR, specifically DQ2, DQ3, DR3, and DR4, as well as HLA-DRB1, to a weakened immune response to the hepatitis B vaccine. Conversely, individuals with type 1 diabetes have shown an increased vaccine response linked to HLA-A11 [48,76].

**Table 2:** Genetic factors affecting hepatitis B vaccine response.

Genetic Factors	Description	Impact	References
HLA Haplotypes	Certain HLA haplotypes display aberrant or deficient immune responses to HBsAg.	Nonresponsiveness to HBsAg antibody formation	[83]
MHC-Linked Genes	Antibody responses to HBV S and pre-S regions are influenced by MHC-linked genes.	Reduced HBV vaccine immunogenicity	[84]
CCR5Δ32	Genetic variant linked to reduced HBV vaccine immunogenicity.	Reduced vaccine effectiveness	[85]
GWAS-Identified Variants	rs1015166, rs12527394, rs2395179, rs3135363, rs3132969, rs34039593, rs35953215, rs3830066, rs4248166, rs477515, rs7745040, rs7770370, rs9267665, rs9268202, rs9268831, rs9273062, rs9277176, rs9277356, rs9277535, rs9277464, and rs9277549.	Positive association with vaccine response	[86]
HLA-DQ Haplotypes	DQ2 and DQ3.	Weakened immune response	[48,76]
HLA-DR Haplotypes	DR3, DR4, and DRB1.	Weakened immune response	[48,76]
HLA-A11	Increased vaccine response linked to HLA-A11 in individuals with type 1 diabetes.	Increased vaccine response	[48,76]

## 4. Future Perspectives

Despite significant advancements in understanding and improving the hepatitis B vaccine response, the landscape of hepatitis B vaccine research is still evolving, offering avenues for further exploration to enhance vaccine efficacy, particularly among nonresponders. Personalized vaccination tailored to the genetic and immunological profiles of individuals holds promise for optimizing vaccine response. Incorporating factors such as age, gender, and underlying medical conditions in personalized vaccination regimens has the potential to optimize immune responses and improve vaccine effectiveness. This approach could involve adjusting vaccine dosages, schedules, or the use of adjuvants to enhance immunogenicity in populations at risk of nonresponse. Advancements in biotechnology can facilitate the creation of next-generation hepatitis B vaccines, including novel adjuvants or alternative delivery systems like intradermal injections or microneedle patches, which could improve efficacy, particularly in those with compromised immune systems or chronic health conditions. Immunomodulatory interventions targeting immune pathways, such as cytokines or immune checkpoint inhibitors, are another promising area of research for enhancing vaccine response.

Long-term immune monitoring is another crucial aspect of future research. The continuous surveillance of vaccine recipients, particularly nonresponders, is essential for assessing the durability of vaccine-induced immunity and the potential need for booster doses. Understanding the mechanisms of waning immunity can inform guidelines for booster vaccinations and long-term hepatitis B prevention strategies. Integrating hepatitis B vaccination efforts into broader public health initiatives can improve the overall vaccine uptake and effectiveness. Strategies such as routine adult vaccination programs, especially for high-risk groups, and increased public awareness campaigns on the importance of hepatitis B vaccination, HBV infection risk, and adherence to vaccination schedules are crucial. Additionally, improving access to healthcare services and vaccination in underserved areas can mitigate the disparities in vaccine response rates. By addressing these issues, future research and public health initiatives can significantly improve the effectiveness of hepatitis B vaccination programs, enhance individual protection, and contribute to the broader goal of global hepatitis B eradication.

## 5. Conclusions

As HBV infection remains a significant health concern globally, understanding and addressing the factors contributing to hepatitis B vaccine nonresponse is crucial for improving the vaccine’s efficacy and necessitates a multifaceted approach. This includes addressing vaccine hesitancy, raising public awareness of the vaccine’s significance, and the use of genetic testing for personalized vaccination protocols. Other strategies include using vaccines with improved immunogenicity, revaccinating or increasing the dose, enhancing adjuvants, implementing different vaccination routes, and co-administering the vaccine with specific drugs.

## Figures and Tables

**Table 1:** Summary factors associated with hepatitis B vaccine nonresponse.

Factor	Effect	Possible Mechanisms	References
Age	Increased nonresponse in individuals aged ≥40 years.	Immunosenescence, decreased thymic function; impaired T-cell and B cell responses; and reduced CD62L and CD28 expression.	[18,19,20]
Gender	Males have higher nonresponse rates than females.	Hormonal differences (testosterone vs. estrogen); genetic variations in immune response genes; and stronger immune memory in females.	[20,21,22]
Obesity	Higher rates of nonresponse in obese individuals.	Vaccine distribution in fat tissue; systemic and B cell inflammation; leptin-induced immune impairment; and vitamin D deficiency.	[23,24,25]
Smoking	Smokers showed reduced vaccine response.	Nicotine-induced T-cell dysfunction; impaired antigen-mediated pathways; and reduced calcium responses in cells.	[26,27]
Alcohol Consumption	Chronic consumption impairs the vaccine’s response; moderate consumption may enhance it.	Suppressed B cells, T-cells, and macrophage functions; reduced responsiveness of B cells to cytokines (IL-2 and IL-4); increased viral replication; oxidative stress; and cytotoxicity.	[28,29,30,31,32]
Diabetes	Lower seroprotection rates among individuals with diabetes.	Defects in antigen presentation; reduced helper T-cells; and genetic predispositions.	[33,34,35]
Chronic Kidney Disease (CKD)	Lower response in patients with CKD, especially in advanced stages.	Immunosuppressive effects of uremia; vitamin D deficiency; hypoalbuminemia; and inflammation.	[36,37,38]
Chronic Liver Disease (CLD)	Reduced vaccine response, especially in cirrhosis and NAFLD.	Immune dysfunction in cirrhosis; impaired T-cell and B cell responses.	[39,40,41]
Chronic Hepatitis C Virus (HCV)	Suboptimal vaccine response in HCV-infected individuals.	Up-regulation of PD-1; overexpression of the killer cell lectin-like receptor sub-family G member 1 (KLRG1); impaired T-cell and humoral immunity.	[42,43,44]
Human Immunodeficiency Virus (HIV)	Wide variability in seroconversion rates based on immune status.	Correlation with CD4+ count; improved response with HAART; and higher seroconversion with CD4+ > 500 cells/mm^3^.	[45,46,47]
Celiac Disease (CD)	Poor response in individuals with CD.	Genetic predisposition (HLA-DQ2) and gluten intake during vaccination.	[48,49,50]
Inflammatory Bowel Disease (IBD)	Reduced response, especially with immunosuppressive treatment.	Malnutrition; immune suppression from biologics and immunomodulators; and impact of TNF-α on B cell function.	[51,52,53]
Genetic Factors	Certain HLA haplotypes linked to nonresponsiveness.	Aberrant or deficient immune responses to HBsAg due to specific genetic profiles.	[54,55]

## Data Availability

Not applicable.
